# Symmetrical and unsymmetrical *α*,*ω*-nucleobase amide-conjugated systems

**DOI:** 10.3762/bjoc.6.34

**Published:** 2010-04-12

**Authors:** Sławomir Boncel, Maciej Mączka, Krzysztof K K Koziol, Radosław Motyka, Krzysztof Z Walczak

**Affiliations:** 1 Silesian University of Technology, Department of Organic Chemistry, Biochemistry and Biotechnology, Krzywoustego 4, 44-100 Gliwice, Poland, Tel.: +48 32 237 1792, Fax: +48 32 237 2094; 2 University of Cambridge, Department of Materials Science and Metallurgy, Pembroke Street, Cambridge CB2 3QZ, United Kingdom, Tel.: +44 1223 334 356, Fax: +44 1223 334 567

**Keywords:** amides, antiprotozoal agents, coupling, nucleosides, self-assembly

## Abstract

We present the synthesis and selected physicochemical properties of several novel symmetrical and unsymmetrical *α*,*ω*-nucleobase mono- and bis-amide conjugated systems containing aliphatic, aromatic or saccharidic linkages. The final stage of the synthesis involves condensation of a subunit bearing carboxylic group with an amine subunit. 4-(4,6-Dimethoxy-1,3,5-triazin-2-yl)-4-methylmorpholinium chloride (DMT-MM) was found to be a particularly effective condensing agent. The subunits containing carboxylic groups were obtained by acidic hydrolysis of *N*-1 Michael adducts of uracils or *N*-9 Michael adducts of 6-chloropurine with methyl acrylate. The amines used were aliphatic/aromatic diamines, adenine, 5-substituted 1-(*ω*-aminoalkyl)uracils and 5′-amino-2′,5′-dideoxythymidine. The title compounds may find application as antiprotozoal agents. Moreover, preliminary microscopy TEM studies of supramolecular behaviour showed that target molecules with bolaamphiphilic structures were capable of forming highly ordered assemblies, mainly nanofibres.

## Introduction

**Figure 1:**
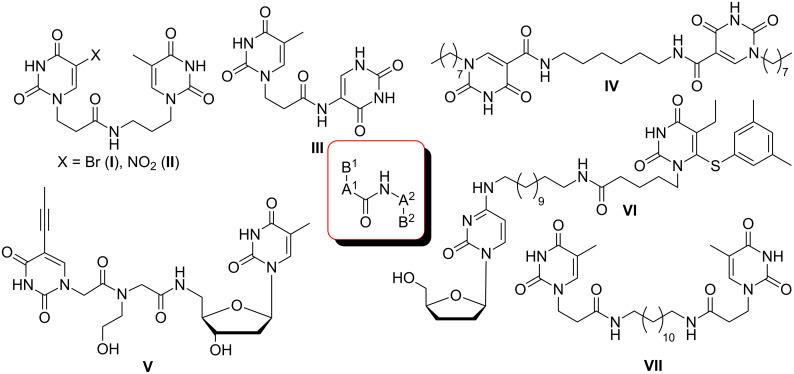
Examples of symmetrical and unsymmetrical *α*,*ω*-nucleobase amide-conjugated systems.

There are numerous reports on the synthesis and application of symmetrical and unsymmetrical *α*,*ω*-nucleobase amide-conjugated systems (Figure 1) [1–6]. These molecules are constructed usually via amide bond(s) from two subunits containing nucleobases or their analogues (B^1^, B^2^) bearing various linkages (A^1^, A^2^).

We have previously reported compounds containing 5-substituted uracil-1-yl subunits bound via short (**I**, **II**) or extra-short (**III**) amide-linkages [1]. These compounds exhibited medium antiprotozoal activity; thus **I** and **III** exhibited growth inhibition of parasities – *Leishmania donovani* and *Plasmodium falciparum* – at levels of 21.4 and 30.6%, respectively [2]. Compound **II** reduced the *Plasmodium falciparum* population by only 17.4%, although it was the most active derivative against it within the group tested. Very recently, Accetta et al. reported remarkable symmetrical amide-conjugated bis-*α*,*ω*-uracil based systems (**IV**). These compounds exhibited antiproliferative and erythroid differentiation induction properties towards human chronic myelogenous leukaemia K562 cells [3]. Moreover, *α*,*ω*-nucleobase amide-conjugated molecules possess the tendency to form meta-stable complexes with DNA and thus can perturb the cell replication. For example, amide-linked heterodimer synthons consisting of acyclic nucleoside units and 5′-amino-2′,5′-dideoxythymidine are PNA/DNA chimeras (**V**) [4]. Furthermore, amide-conjugated systems of solely biologically active units may be utilised in combined anti-HIV therapy, e.g. 2′,3′-dideoxycytidine and 1-[(2-hydroxyethoxy)methyl]-6-(phenylthio)thymine conjugate (ddC-HEPT) (**VI**) [5].

In addition, Shimizu et al. in 2001 reported structures, which are formally *α*,*ω*-nucleobase bolaamphiphiles (**VII**), capable of forming extraordinary nanotopologies [6].

In order to synthesise a new group of modified, mainly pyrimidinic nucleosides, we employed a simple and effective synthetic pathway based on the catalytic aminolysis of carboxylic acids. Our synthetic approach surpasses all the procedures previously reported, not only in terms of higher yields, even although earlier reports of the synthesis of symmetrical *α*,*ω*-nucleobase amide-conjugated systems were based on a similar strategy. These methods involved two-step procedures such as catalytic condensation, e.g. in the presence of a suitable coupling agent – *N,N*′-dicyclohexylcarbodiimide (DCC) [7], or acylation of appropriate amines with acyl halides [3] or “active esters” [6]. Monoacylation of aliphatic diamines, either in the presence of MgCl_2_ [8–9] or 9-borabicyclo[3.3.1]nonane (9-BBN) [10], has been used for the synthesis of unsymmetrical derivatives. Interestingly, considering the physicochemical aspect of our work, two of the compounds synthesised containing aliphatic long-chain linkages revealed a tendency to self-assemble in the initial TEM studies.

## Results and Discussion

**Scheme 1:**
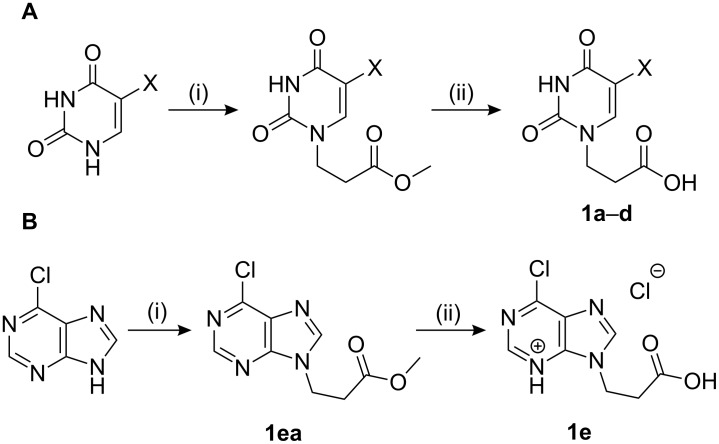
Synthesis of main acidic subunits, precursors of both symmetrical and unsymmetrical *α*,*ω*-nucleobase amide-conjugated systems: **A** – 5-substituted uracil-based [11] – (i) methyl acrylate (2 equiv), TEA (1 equiv), DMF, 20 °C, 24 h, (ii) 5% HCl_aq_, reflux, 0.5 h; **B** – 6-chloropurine-based – (i) methyl acrylate (2 equiv), Hünig’s base (1 equiv), 20 °C, 24 h, (ii) 5% HCl_aq_, reflux, 1 h.

The subunits containing carboxylic groups were obtained by acidic hydrolysis of *N*-1 Michael adducts of uracils or *N*-9 Michael adducts of 6-chloropurine with methyl acrylate (Scheme 1A) [1]. Uracil derivatives were obtained via the fully regioselective one-step procedure as previously reported [11–12]. To construct systems containing modified purinic nucleobase, we synthesised the *N*-9 adduct (**1ea**) of 6-chloropurine with methyl acrylate in 90% yield using Hünig’s base (diisopropylethylamine) as a deprotonating agent (Scheme 1B) (see Supporting Information File 1).

**Figure 2:**
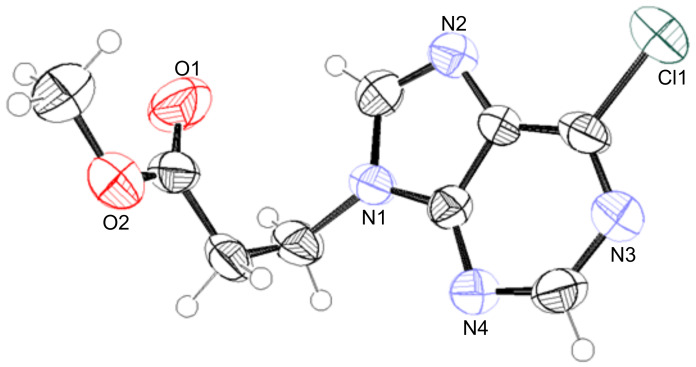
X-ray structure of **1ea** shown as thermal ellipsoids at 50% probability [17–18].

X-ray analysis confirmed the structure of this regioisomer (Figure 2) [13]. This simple method appears to be the most *N*-9 regioselective process compared to other methods [14–16].

The *N*-7 adduct of 6-chloropurine (**1eb**) was also isolated in 10% yield. Hydrolysis of the *N*-9 adduct under acidic conditions (5% HCl_aq_) gave 9-(2-carboxyethyl)-6-chloro-9*H*-purinium-3 chloride (**1e**). As amine components, readily accessible amines such as aliphatic *α*,*ω*-diamines, phenylene diamines, adenine, 5-substituted 1-(*ω*-aminoalkyl)uracils and 5′-amino-5′-deoxythymidine were chosen.

**Figure 3:**
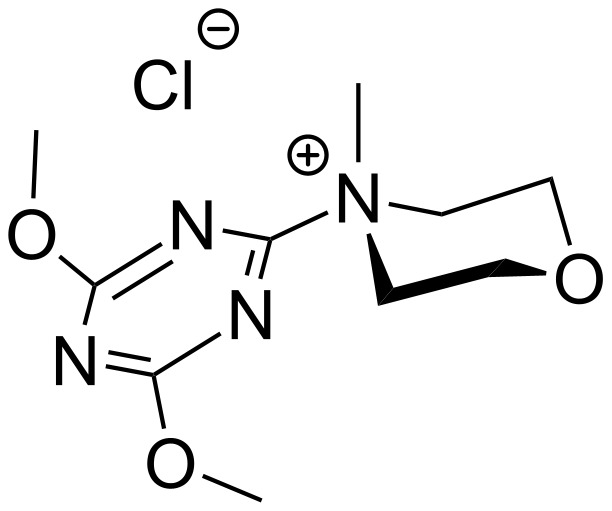
DMT-MM in its presumably more stable conformation [20].

To accomplish the synthesis of the title compounds, we developed a simple and effective method of coupling the carboxylic subunit with an amino compound. As condensing agent, 4-(4,6-dimethoxy-1,3,5-triazin-2-yl)-4-methylmorpholinium chloride (DMT-MM) was found to be very convenient and efficient (Figure 3) [19].

We have previously used this catalyst successfully in our search for unsymmetrical *α*,*ω*-nucleobase molecules, which are structurally related to the title compounds, i.e. short linkage amide- [1] and ester-conjugated systems [12].

### Symmetrical *α*,*ω*-nucleobase amide-conjugated systems

The first set of compounds – symmetrical aliphatic and aromatic *α*,*ω*-nucleobase amide-conjugated systems (**4**–**15**, Table 1) – was synthesised from 3-(5-substituted-uracil-1-yl)propionic acids **1a**–**d** and the appropriate aliphatic *α*,*ω*-diamines **2a**–**e**, and the aromatic diamines **3a**–**c** in DMF (see Supporting Information File 1).

**Table 1:** Symmetrical aliphatic and aromatic *α*,*ω*-nucleobase amide-conjugated systems.

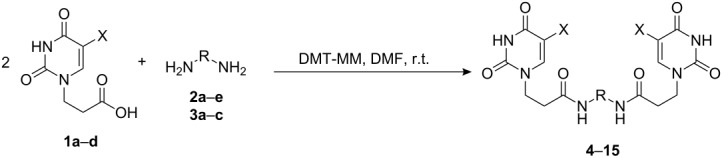					
Acidic subunit	X	Amine subunit	R	Product	Yield [%]

**1c**	Br	**2a**	(CH_2_)_2_	**4**	57
**1a**	H	**2b**	(CH_2_)_3_	**5**	64
**1b**	CH_3_	**2b**	(CH_2_)_3_	**6**	60
**1d**	NO_2_	**2b**	(CH_2_)_3_	**7**	55
**1b**	CH_3_	**2c**	(CH_2_)_6_	**8**	67
**1c**	Br	**2c**	(CH_2_)_6_	**9**	31
**1a**	H	**2d**	(CH_2_)_9_	**10**	74
**1b**	CH_3_	**2e**	(CH_2_)_10_	**11**	59
**1d**	NO_2_	**2e**	(CH_2_)_10_	**12**	67
**1c**	Br	**3a**	*o*-phenylene	**13**	35
**1c**	Br	**3b**	*m*-phenylene	**14**	37
**1a**	H	**3c**	*p*-phenylene	**15**	68

The yields vary from moderate to good. The lower yields noted in the table arise as a result of the method of isolation (see Supporting Information File 1). Despite its susceptibility to nucleophilic attack, 5-nitrouracil acid derivative **1d** also reacted with aliphatic diamines and gave the expected diamides **7** and **12**.

### Unsymmetrical *α*,*ω*-nucleobase amide-conjugated systems

The second group – unsymmetrical *α*,*ω*-nucleobase amide-conjugated systems and their synthetic precursors (Table 2) – was synthesised from monoacid and monoamine derivatives with the exception of mono-amine precursor **16** which was synthesised from 1,6-hexylenediamine.

This synthesis was based on the classical approach of half-conversion of the bifunctional reactant with the monofunctional compound by using an excess of diamine (3 equiv) with respect to the acidic reactant. Nevertheless, this procedure led to problems in the purification step and partial decomposition of DMT-MM during the reaction. This was especially significant when compared to the synthesis of *N*-Boc monoprotected amine reactant **17** (See Supporting Information File 1).

The compounds in this second group were obtained in yields of 31–95%. The synthesis of nucleosides containing aliphatic linkages (**19**–**22**) presented no difficulties on work-up.

**Table 2:** Unsymmetrical *α*,*ω*-nucleobase amide-conjugated systems based on aliphatic or saccharidic linkages and their exemplary synthetic precursors.

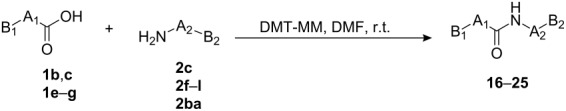						
B^1^A^1^–**COOH**		**H**_2_**N**–A^2^B^2^		Pro- duct	B^1^A^1^−**CONH**−A^2^B^2^	Yield [%]

**1c**	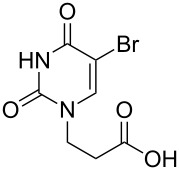	**2c**	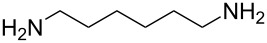	**16**	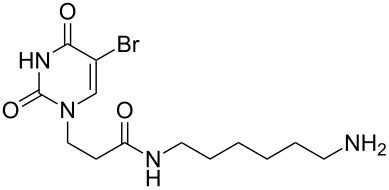	33
**1b**	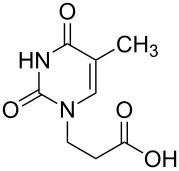	**2ba**	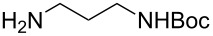	**17**	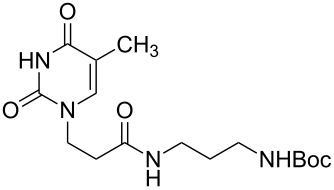	99
**1b**	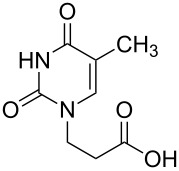	**2f**	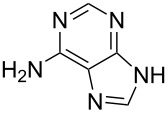	**18**	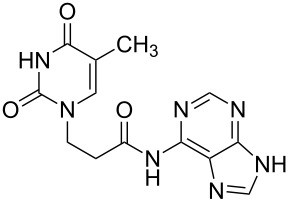	68
**1c**	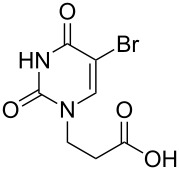	**2g**	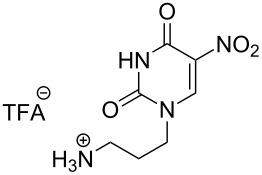	**19**	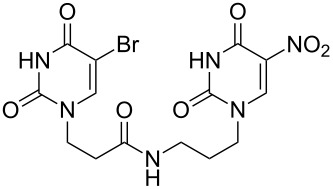	90
**1c**	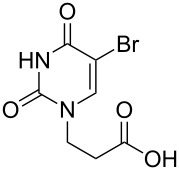	**2h**	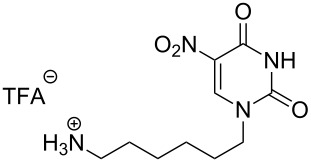	**20**	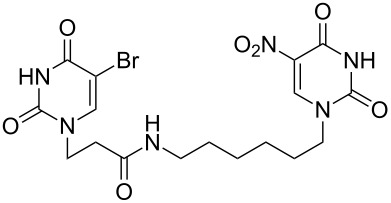	83
**1e**	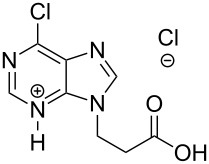	**2i**	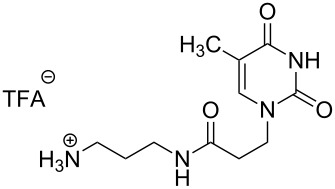	**21**	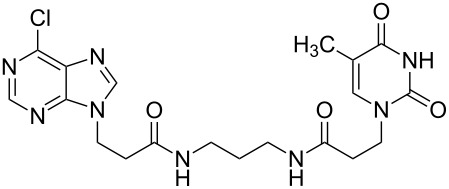	95
**1e**	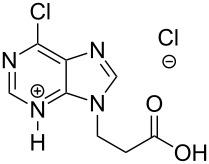	**2j**	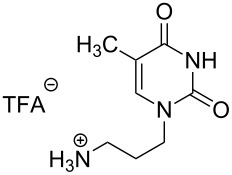	**22**	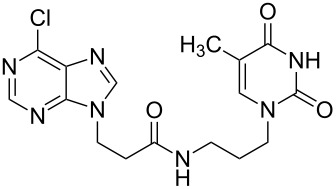	44
**1f**	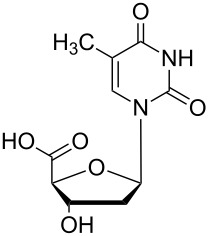	**2h**	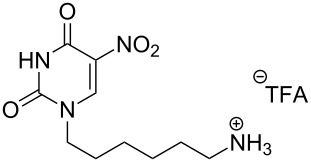	**23**	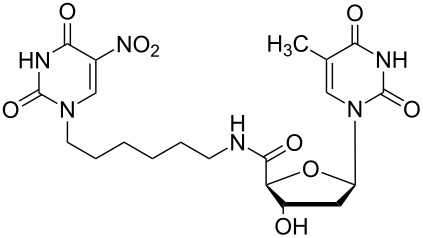	85
**1g**	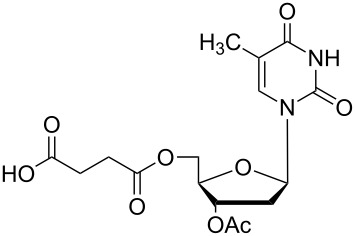	**2k**	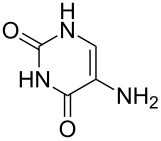	**24**	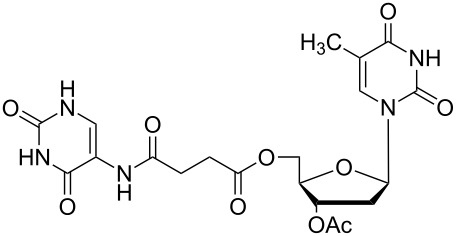	31
**1c**	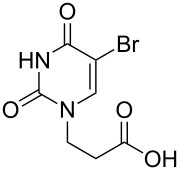	**2l**	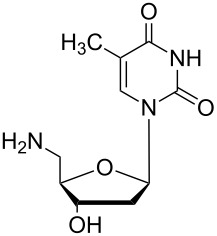	**25**	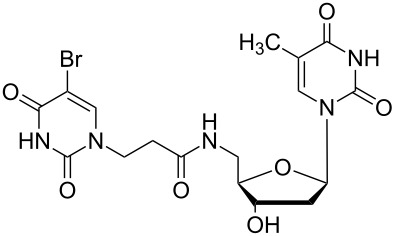	92

Two different acidic and three different amine subunits – synthesised according to known procedures – were utilised in the synthesis of **19**, **20** and **23**–**25**. The acidic subunits used were (2*S*,3*S*,5*R*)-3-hydroxy-5-[5-methyl-2,4-dioxo-3,4-dihydropyrimidin-1(2*H*)-yl]tetrahydrofuran-2-carboxylic acid (**1f**) [21] and 4-({(2*R*,3*S*,5*R*)-3-acetoxy-5-[5-methyl-2,4-dioxo-3,4-dihydropyrimidin-1(2*H*)-yl]tetrahydro-furan-2-yl}methoxy)-4-oxobutanoic acid (**1g**) [22]. The amine subunits employed were 3-[5-nitro-2,4-dioxo-3,4-dihydropyrimidin-1(2*H*)-yl]propan-1-aminium trifluoroacetate (**2g**) [23], 6-[5-nitro-2,4-dioxo-3,4-dihydropyrimidin-1(2*H*)-yl]hexan-1-aminium trifluoroacetate (**2h**) [23] and 1-[(2*R*,4*S*,5*R*)-5-(aminomethyl)-4-hydroxytetrahydrofuran-2-yl]-5-methylpyrimidine-2,4-(1*H*,3*H*)-dione (**2l**) [24]. The synthesis of the amine subunit **2j** was carried out according to the known procedure [25]. The unprotected hydroxyl group in sugar moieties, as expected, was entirely unreactive towards the carboxylic group when compared to the amine functionality, and consequently nucleosides with a saccharide moiety (**23**, **25**) were successfully synthesised.

### Self-assembly of aliphatic long-chain symmetrical *α*,*ω*-nucleobase amide-conjugated systems

**Figure 4:**
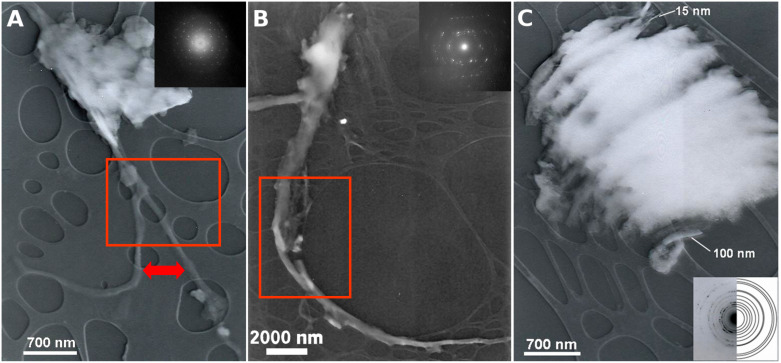
TEM images of symmetrical *α*,*ω*-nucleobase amide-conjugated systems: **A** – two splitting nucleoside nanofibres growing out from crystalline phase (**11**), the rectangular area (**A**) shows “twin fibres”; **B** – “twin fibres” growing out from microcrystalline “lizard tail” (**11**); **C** – nanofibres in bulky microcrystalline area (**12**).

For the microscopic studies, we selected the two model compounds **11** and **12**. The supramolecular behaviour of conjugated nucleobase-based bolaamphiphiles was examined by TEM. A JEOL 200 FX, operating at 200 kV, was used to obtain the typical images as shown in Figure 4. For the microscopic studies, saturated ethanolic solutions of **11** and **12** prepared by ultrasonication were subjected to slow vaporisation at −20 °C. Microcrystalline assemblies were grown directly on copper grids for TEM analyses. This procedure of growth accompanied by careful investigations revealed that thymine- (**A**, **B**) and 5-nitrouracil-based (**C**) bolaamphiphiles derived from 1,10-diaminodecane may form nanofibres of 80–300 nm diameter for thymine and 15–100 nm for 5-nitrouracil derivatives, respectively.

**Figure 5:**
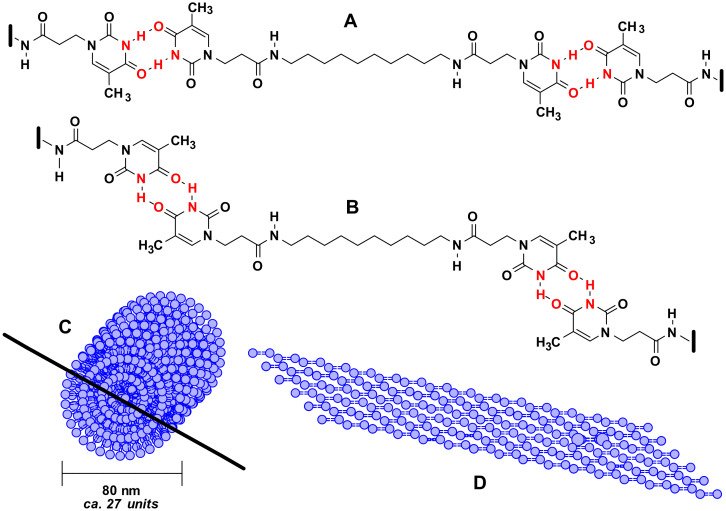
Models of micro- and nanofibres based on hydrogen bonding interactions between thymine units; (**A**) thermodynamically favourable linear system of hydrogen bonds; (**B**) less favourable “bent” system of hydrogen bonds; (**C**) **π**-stacking and hydrogen bonding based nanofibre; (**D**) longitudinal cross-section of a particular nanofibre.

The electron diffraction patterns in the case of **11** indicate single crystal fibre diffraction (Figure 4A). A simplified model for the formation of multilayered nanofibres is shown in Figure 5.

A proposal of simplified models of micro- and nanofibres is based on the π-stacking and hydrogen bonding interactions between thymine units. Amide bonds, on the other hand, may be crucial in the inter-layer hydrogen bonding. A linear system of hydrogen bonding appears as a thermodynamically favourable (**A**). Nevertheless, a less favourable “bent” (or “wobble”) system could also be involved [26]. This fact is reflected in the presence of residual electron diffraction patterns, which are in particular visible in the case of so-called “twin fibres” (**B**) growing out from the microcrystalline phase – “lizard tail”. A complete model of the nanofibre is presented in Figure 5C along with its longitudinal cross-section (**D**).

## Conclusion

We have synthesised a novel group of twelve symmetrical and ten unsymmetrical *α*,*ω*-nucleobase amide-conjugated systems using DMT-MM as the condensing agent. These two groups of compounds will be investigated for antiprotozoal activity within the framework of the Drugs for Neglected Diseases initiative (DND*i*). These investigations are currently in progress.

Additionally, we have undertaken preliminary TEM studies on the self-assembly of two symmetrical aliphatic long-chain compounds. Their supramolecular nature was revealed since they form nanofibres during low temperature crystal growth. These experiments were reproducible, but further studies are required.

## Supporting Information

Experimental Section

File 1 The data provides general remarks, procedures, physical properties and ^1^H and ^13^C NMR spectra of all newly synthesised compounds.
