# Supplementary material for: Symmetrical and unsymmetrical α,ω-nucleobase amide-conjugated systems
Source: Beilstein J Org Chem. 2010 Apr 12;6:34. doi: 10.3762/bjoc.6.34 (PMC2874405; doi:10.3762/bjoc.6.34)
Supplement: File 1 — The data provides general remarks, procedures, physical properties and 1H and 13C NMR spectra of all newly synthesised compounds. [file Beilstein_J_Org_Chem-06-34-s001.pdf]

Supporting Information
for

Symmetrical and unsymmetrical *α,ω*-nucleobase amide-conjugated systems

Sławomir Boncel[1], Maciej Mączka[1], Krzysztof K. K. Koziol[2], Radosław Motyka[1] and Krzysztof Z. Walczak*[1]

[1]Silesian University of Technology, Department of Organic Chemistry, Biochemistry and Biotechnology, Krzywoustego 4, 44-100 Gliwice, Poland, Tel.: +48 32 237 1792, Fax: +48 32 237 2094 and [2]University of Cambridge, Department of Materials Science and Metallurgy, Pembroke Street, Cambridge CB2 3QZ, United Kingdom, Tel.: +44 1223 334 356, Fax: +44 1223 334 567

E-mail: Krzysztof Z. Walczak - krzysztof.walczak@polsl.pl

* Corresponding author

Experimental Section

General: NMR spectra were recorded at 300 MHz for ^{1}H NMR and 75.5 MHz for ^{13}C NMR on a Varian Inova 300 MHz in [D_6]DMSO solution; δ values are in parts per million relative to tetramethylsilane as an internal standard. Elemental analyses were obtained using a Perkin-Elmer 240C apparatus. IR spectra were recorded on a Perkin IR-Spectrometer. Mass spectra were recorded at ESI TOF ionisation on Ionspec 4.7 Tesla Ultima FTMS FT-ICR MS Fourier Transform Ion Cyclotron Resonance Mass Spectrometer. X-ray diffraction data were collected on KUMA KM4 four-circle diffractometer at 295 K. Cell parameters, reflection collection and their reductions were done using KUMA KM4 software. The structures were determined by means of direct methods and refined by the full-matrix least squares technique . TEM analyses were conducted on JEOL 200 CX operating at 120 kV in bright/dark field and diffraction modes. All used reagents were purchased from Aldrich. TLC $60F_{254}$ plates and silica gel 60 (0.040–0.063 mm) were purchased from Merck (also diamines) or from Alfa Aesar. Melting points were measured at Boetius apparatus and are uncorrected. DMF was distilled prior to use and stored over molecular sieves 4 Å.

Michael-type adducts of 6-chloropurine to methyl acrylate

To a solution of 6-chloropurine (5 mmol) in anhydrous DMF (10 ml), Hünig's base (5 mmol) was added with stirring. After 5 minutes methyl acrylate (15 mmol) was added dropwise. Stirring was continued and when the consumption of the limiting reactant was achieved (TLC, 5% MeOH/$CHCl_3$, vol.), the solvent was evaporated under reduced pressure and the residue purified on a chromatographic column using 5% MeOH/$CHCl_3$ (vol.) as eluant.

Methyl 3-(6-chloro-9*H*-purin-9-yl)propanoate (1ea)

Mp 83–84 °C. ^{1}H NMR: δ = 8.77 (s, H-2, 1 H), 8.70 (s, H-8, 1 H), 4.56 (t, $^3J_1$ = 6.6 Hz, >NCH_2, 2 H), 3.59 (s, OCH_3, 3 H), 3.06 (t, $^3J_1$ = 6.6 Hz, CH_2CO, 2 H). ^{13}C NMR: δ = 170.9, 151.9, 151.4, 148.9, 147.7, 130.8, 51.7, 39.7, 32.9. $C_9H_9ClN_4O_2$ (240.65): calcd. C 44.92, H 3.77, N 23.28; found C 44.77, H 3.92, N 23.15.

Methyl 3-(6-chloro-7*H*-purin-7-yl)propanoate (1eb)

Mp 81–82 °C. ^{1}H NMR: δ = 8.78 (m, H-2, H-8, 2 H), 4.72 (t, $^3J_1$ = 6.6 Hz, >NCH_2, 2 H), 3.57 (s, OCH_3, 3 H), 3.04 (t, $^3J_1$ = 6.6 Hz, CH_2CO, 2 H). ^{13}C NMR: δ = 170.8, 151.5, 151.3, 148.9, 122.1, 42.1, 39.8, 34.8. $C_9H_9ClN_4O_2$ (240.65): calcd. C 44.92, H 3.77, N 23.28; found C 45.09, H 3.59, N 23.16.

Symmetrical *α,ω*-nucleobase amide-conjugated systems

General procedure

To a stirred solution (at room temperature) of the appropriate acidic component (**1a–d**) (0.5 mmol) in anhydrous DMF (8 mL), diamine (1 mmol) was added. In the case of synthesis of **4**, when the diamine hydrochloride was used, 1 mmol of *N*-methylmorpholine was added to liberate the free amine. After 10 minutes to the clear solution (in the synthesis of **8–12**) or the suspension (**4–7** and **13–15**), DMT-MM (1 mmol) was added. The progress of reaction was monitored by TLC (20% vol. MeOH/$CHCl_3$). After 1.5–6 h the volatiles were removed under reduced pressure and the residue purified by gradient column chromatography (0–50% vol. MeOH/$CHCl_3$) or recrystallised from ethanol/water (**4–7** and **13–15**) or ethyl acetate/hexane (**8–12**). All the obtained compounds were white solids unless otherwise indicated.

***N,N*'-(ethane-1,2-diyl)bis{3-[5-bromo-2,4-dioxo-3,4-dihydropyrimidin-1(2*H*)-yl]propanamide} (4)**

Mp 291–294 °C. ^{1}H NMR: δ = 11.75 (s, H-3, 2 H), 8.09 (s, H-6, 2 H), 8.02 (br s, CONH, 2 H), 3.85 (t, $^3J_1$ = 6.6 Hz, 4 H, >NCH_2), 3.05 (m, 4 H, $NHCH_2CH_2NH$), 2.45 (t, $^3J_1$ = 6.6 Hz, 4 H, CH_2CO). ^{13}C NMR: δ = 169.7 (2 C), 159.7 (2 C), 150.2 (2 C), 145.9 (2 C), 94.1 (2 C), 45.0 (2 C), 38.3 (2 C), 34.0 (2 C). $C_{16}H_{18}Br_2N_6O_6$ (550.16): calcd. C 34.93, H 3.30, N 15.28; found C 34.78, H 3.39, N 15.33.

***N,N*'-(propane-1,3-diyl)bis{3-[2,4-dioxo-3,4-dihydropyrimidin-1(2*H*)-yl]propanamide} (5)**

Mp 278–279 °C. ^{1}H NMR: δ = 11.25 (br s, H-3, 2 H), 7.96 (br s, CONH, 2 H), 7.52 (d, ^3J = 7.2 Hz, H-6, 2 H), 5.50 (d, ^3J = 7.2 Hz, H-5, 2 H), 3.84 (t, ^3J = 6.6 Hz, 4 H, >NCH_2), 3.01 (quartet, ^3J = 6.6 Hz, 4 H, $CONHCH_2$), 2.44 (t, ^3J = 6.6 Hz, 4 H, CH_2CO), 1.47 (m, $CH_2CH_2CH_2$, 2 H). ^{13}C NMR: δ =169.5 (2 C), 163.8 (2 C), 150.8 (2 C), 146.2 (2 C), 100.5 (2

C), 44.8 (2 C), 36.4 (2 C), 34.2 (2 C), 29.1. $C_{17}H_{22}N_6O_6$ (406.39): calcd. C 50.24, H 5.46, N 20.68; found C 50.19, H 5.46, N 20.71.

***N,N'*-(propane-1,3-diyl)bis{3-[5-methyl-2,4-dioxo-3,4-dihydropyrimidin-1(2*H*)-yl]propanamide} (6)**

Mp 271–273 °C. ^{1}H NMR: δ = 11.22 (s, H-3, 2 H), 7.94 (t, ^3J = 6.6 Hz, CONH, 2 H), 7.39 (d, ^4J = 0.6 Hz, H-6, 2 H), 3.81 (t, ^3J = 6.6 Hz, >NCH_2, 4 H), 3.01 (quartet, ^3J = 6.6 Hz, 4 H, CONHCH_2), 2.42 (t, ^3J = 6.6 Hz, 4 H, CH_2CO), 1.71, (d, ^4J = 0.6 Hz, CH_3, 6 H), 1.44 (quintet, ^3J = 6.6 Hz, $CH_2CH_2CH_2$, 2 H). ^{13}C NMR: δ = 169.5 (2 C), 164.3 (2 C), 150.7 (2 C), 141.9 (2 C), 108.0 (2 C), 44.6 (2 C), 36.3 (2 C), 34.4 (2 C), 29.2, 11.9 (2 C). $C_{19}H_{26}N_6O_6$ (434.45): calcd. C 52.53, H 6.03, N 19.34; found C 52.42, H 6.03, N 19.18.

***N,N'*-(propane-1,3-diyl)bis{3-[5-nitro-2,4-dioxo-3,4-dihydropyrimidin-1(2*H*)-yl]propanamide} (7)**

Mp 144–147 °C. ^{1}H NMR: δ = 11.25 (s, H-3, 2 H), 9.12 (s, H-6, 2 H), 8.02 (t, ^3J = 5.4 Hz, CONH, 2 H), 4.07 (t, ^3J = 6.6 Hz, >NCH_2, 4 H), 3.03 (quartet, ^3J = 6.3 Hz, 4 H, CONHCH_2), 2.55 (t, ^3J = 6.6 Hz, 4 H, CH_2CO), 1.48 (quintet, ^3J = 6.3 Hz, $CH_2CH_2CH_2$, 2 H). ^{13}C NMR: δ = 169.5 (2 C), 155.2 (2 C), 151.4 (2 C), 149.4 (2 C), 124.5 (2 C), 46.3 (2 C), 36.4 (2 C), 33.6 (2 C), 29.1. $C_{17}H_{20}N_8O_{10}$ (496.39): calcd. C 41.13, H 4.06, N 22.57; found C 40.95, H 3.98, N 22.40.

***N,N'*-(hexane-1,6-diyl)bis{3-[5-methyl-2,4-dioxo-3,4-dihydropyrimidin-1(2*H*)-yl]propanamide} (8)**

Mp 238–239 °C. ^{1}H NMR: δ = 11.23 (s, H-3, 2 H), 7.94 (t, ^3J = 6.6 Hz, CONH, 2 H), 7.39 (d, ^4J = 0.6 Hz, H-6, 2 H), 3.82 (t, ^3J = 6.6 Hz, >NCH_2, 4 H), 3.00 (quartet, ^3J = 6.6 Hz, 4 H, CONHCH_2), 2.42 (t, ^3J = 6.6 Hz, 4 H, CH_2CO), 1.72 (d, ^4J = 0.6 Hz, CH_3, 6 H), 1.40–1.24 (m, $NHCH_2CH_2$, 4 H), 1.26–1.10 (m, $NHCH_2CH_2CH_2$, 4 H). ^{13}C NMR: δ = 169.3 (2 C), 164.3 (2 C), 150.7 (2 C), 141.9 (2 C), 107.9 (2 C), 44.6 (2 C), 38.4 (2 C), 34.3 (2 C), 29.0 (2 C), 26.0 (2 C), 11.9 (2 C). ESI MS (*m/z*): M^+ + Na = 499.23 (100%), 500.26 (78%); calcd. M^+ + Na = 499.53. IR (KBr): ν = 3340, 3161, 3040, 2928, 1695, 1674, 1550, 1467, 1456, 1348, 1217 cm^{-1}. $C_{22}H_{32}N_6O_6$ (476.53): calcd. C 55.45, H 6.77, N 17.64; found C 55.56, H 6.64, N 17.86.

***N*,*N'*-(hexane-1,6-diyl)bis{3-[5-bromo-2,4-dioxo-3,4-dihydropyrimidin-1(2*H*)-yl]propanamide} (9)**

Mp 223–224 °C. ^{1}H NMR: δ = 11.76 (s, H-3, 2 H), 8.03 (s, H-6, 2 H), 7.95 (t, ^{3}J = 6.6 Hz, CONH, 2 H), 3.88 (t, ^{3}J = 6.6 Hz, >NCH_2, 4 H), 3.00 (quartet, ^{3}J = 6.6 Hz, CONHCH_2, 4 H), 2.45 (t, ^{3}J = 6.6 Hz, CH_2CO, 4 H), 1.40–1.26 (m, $NHCH_2CH_2$, 4 H), 1.24–1.10 (m, $NHCH_2CH_2CH_2$, 4 H). ^{13}C NMR: δ = 169.2 (2 C), 159.7 (2 C), 150.1 (2 C), 145.9 (2 C), 94.0 (2 C), 45.2 (2 C), 38.4 (2 C), 34.0 (2 C), 29.0 (2 C), 26.1 (2 C). ESI MS (*m/z*): M^+ + Na = 629.00 (100%), 627.01 (78%), 631.01 (60%); calcd. M^+ + Na = 629.26. $C_{20}H_{26}Br_2N_6O_6$ (606,27): calcd. C 39.62, H 4.32, N 13.86; found C 39.51, H 4.34, N 13.95.

***N*,*N'*-(nonane-1,9-diyl)bis{3-[2,4-dioxo-3,4-dihydropyrimidin-1(2*H*)-yl]propanamide} (10)**

Mp 117–118 °C. ^{1}H NMR: δ = 11.24 (s, H-3, 2 H), 7.94 (t, ^{3}J = 6.6 Hz, CONH, 2 H), 7.51 (d, ^{3}J = 7.8 Hz, H-6, 2 H), 5.48 (dd, $^{3}J_1$ = 7.8 Hz, $^{4}J_2$ = 1.8 Hz, H-5, 2 H), 3.84 (t, ^{3}J = 6.6 Hz, >NCH_2, 4 H), 3.00 (quartet, ^{3}J = 6.6 Hz, CONHC*H*2, 4 H), 2.43 (t, ^{3}J = 6.6 Hz, 4 H, CH_2CO), 1.42–1.28 (m, $NHCH_2CH_2$, 4 H), 1.28–1.12 (m, $(CH_2)_5$, 10 H). ^{13}C NMR: δ = 169.2 (2 C), 163.8 (2 C), 150.8 (2 C), 146.2 (2 C), 100.4 (2 C), 44.8 (2 C), 38.4 (2 C), 34.2 (2 C), 29.0 (2 C), 28.9, 28.7 (2 C), 26.4 (2 C). $C_{23}H_{34}N_6O_6$ (490.55): calcd; C 56.31, H 6.99, N 17.13; found C 56.49, H 7.08, N 17.25.

***N*,*N'*-(decane-1,10-diyl)bis{3-[5-methyl-2,4-dioxo-3,4-dihydropyrimidin-1(2*H*)-yl]propanamide} (11)**

Mp 214–216 °C. ^{1}H NMR: δ = 11.22 (s, H-3, 2 H), 7.94 (t, ^{3}J = 6.6 Hz, CONH, 2 H), 7.37 (s, H-6, 2 H), 3.81 (t, ^{3}J = 6.6 Hz, >NCH_2, 4 H), 3.00 (quartet, ^{3}J = 6.6 Hz, 4 H, CONHCH_2), 2.41 (t, ^{3}J = 6.6 Hz, CH_2CO, 4 H), 1.72 (s, CH_3, 6 H), 1.49–1.23 (m, $NHCH_2CH_2$, 4 H), 1.23–1.11 (m, $(CH_2)_6$, 12 H). ^{13}C NMR: δ = 169.2 (2 C), 164.3 (2 C), 150.7 (2 C), 141.9 (2 C), 107.9 (2 C), 44.6 (2 C), 38.4 (2 C), 34.3 (2 C), 29.0 (2 C), 28.9 (2 C), 28.7 (2 C), 26.3 (2 C), 11.9 (2 C). IR (KBr): ν = 3379, 3172, 3041, 2930, 2854, 1699, 1697, 1670, 1550, 1470, 1364, 1217 cm^{-1}. $C_{26}H_{40}N_6O_6$ (532.63): calcd. C 58.63, H 7.57, N 15.78, O 18.02; found C 58.48, H 7.38, N 15.60.

***N,N'*-(decane-1,10-diyl)bis{3-[5-nitro-2,4-dioxo-3,4-dihydropyrimidin-1(2*H*)-yl]propanamide} (12)**

Yellowish solid, mp 218–219 °C. ^{1}H NMR: δ = 12.02 (br s, H-3, 2 H), 9.13 (s, H-6, 2 H), 7.97 (t, ^3J = 6.6 Hz, CONH, 2 H), 4.05 (t, ^3J = 6.6 Hz, >NCH_2, 4 H), 3.00 (quartet, ^3J = 6.6 Hz, 4 H, CONHCH_2), 2.51 (t, ^3J = 6.6 Hz, CH_2CO, 4 H), 1.40–1.26 (m, $NHCH_2CH_2$, 4 H), 1.26–1.12 (m, $(CH_2)_6$, 12 H). ^{13}C NMR: δ = 169.1 (2 C), 154.9 (2 C), 151.2 (2 C), 149.1 (2 C), 124.4 (2 C), 46.2 (2 C), 38.5 (2 C), 33.6 (2 C), 29.0 (2 C), 28.9 (2 C), 28.7 (2 C), 26.4 (2 C). IR (KBr): ν = 3327, 3200, 3087, 2931, 2853, 1728, 1691, 1637, 1620, 1554, 1515, 1465, 1371, 1319, 1271, 1238, 1203 cm^{-1}. $C_{24}H_{34}N_8O_{10}$ (594,57): calcd. C 48.48, H 5.76, N 18.85, O 26.91; found C 48.56, H 5.81, N 19.03.

***N,N'*-(1,2-phenylene)bis{3-[5-bromo-2,4-dioxo-3,4-dihydropyrimidin-1(2*H*)-yl]propanamide} (13)**

Mp 288–289 °C (decomp.). ^{1}H NMR: δ = 11.78 (br s, H-3, 2 H), 9.37 (s, CONH, 2 H), 8.16 (s, H-6, 2 H), 7.51 (dd, $^3J_1$ = 6.0 Hz, $^4J_1$ = 3.3 Hz, *o*-H, 4 H), 7.13 (dd, $^3J_1$ = 6.0 Hz, $^4J_1$ = 3.3 Hz, *m*-H, 4 H), 3.99 (t, ^3J = 6.6 Hz, >NCH_2, 4 H), 2.75 (t, ^3J = 6.6 Hz, CH_2CO, 4 H). ^{13}C NMR: δ = 169.0 (2 C), 162.3 (2 C), 159.7 (2 C), 150.3 (2 C), 145.9 (2 C), 130.2 (2 C), 124.8 (2 C), 94.2 (2 C), 44.9 (2 C), 34.8 (2 C). IR (KBr): ν = 3471, 3251, 3190, 3089, 3047, 2829, 1718, 1708, 1639, 1537, 1535, 1446, 1436, 1353, 1257, 1222, 1217 cm^{-1}. $C_{20}H_{18}Br_2N_6O_6$ (598.20): calcd. C 40.16, H 3.03, N 14.05; found: C 40.08, H 2.92, N 14.07.

***N,N'*-(1,3-phenylene)bis{3-[5-bromo-2,4-dioxo-3,4-dihydropyrimidin-1(2*H*)-yl]propanamide} (14)**

White spherulic crystals, mp 266–267 °C. ^{1}H NMR: δ = 11.78 (br s, H-3, 2 H), 10.08 (s, CONH, 2 H), 8.17 (s, H-6, 2 H), 7.34-7.16 (m, aromatic H, 8 H), 3.97 (t, ^3J = 6.6 Hz, >NCH_2, 4 H), 2.73 (t, ^3J = 6.6 Hz, CH_2CO, 4 H). ^{13}C NMR: δ = 168.7 (2 C), 159.7 (2C), 150.2 (2C), 145.9 (2C), 139.2 (2C), 128.9 (2C), 114.2 (2C), 110.1 (2C), 94.1 (2C), 44.9 (2C), 34.9 (2C). IR (KBr): ν = 3481, 3330, 1690, 1647, 1544, 1452, 1440, 1348 cm^{-1}. $C_{20}H_{18}Br_2N_6O_6$ (598.20): calcd. C 40.16, H 3.03, N 14.05; found: C 40.33, H 3.17, N 13.89.

***N,N*'-(1,4-phenylene)bis{3-[2,4-dioxo-3,4-dihydropyrimidin-1(2*H*)-yl]propanamide} (15)**

Mp > 300 °C. ^{1}H NMR: δ = 11.27 (br d, $^4J_1$ = 1.8 Hz, H-3, 2 H), 9.99 (s, CONH, 2 H), 7.60 (d, ^3J = 7.8 Hz, H-6, 2 H), 7.47 (s, aromatic H, 4 H), 5.51 (dd, $^3J_1$ = 7.8 Hz, $^4J_2$ = 1.8 Hz, H-5,

2 H), 3.94 (t, ^3J = 6.3 Hz, >NCH_2, 4 H), 2.69 (t, ^3J = 6.3 Hz, CH_2CO, 4 H). ^{13}C NMR: δ = 168.4 (2C), 163.8 (2C), 150.9 (2C), 146.3 (2C), 134.4 (2C), 119.6 (2C), 100.5 (2C), 44.7 (2C), 35.0 (2C). IR (KBr): ν = 3463, 3327, 3174, 3051, 1683, 1573, 1517, 1461, 1407, 1309, 1303, 1242 cm^{-1}. $C_{20}H_{20}N_6O_6$ (440.41): calcd. C 54.54, H 4.58, N 19.08; found C 54.59, H 4.37, N 19.02.

Unsymmetrical *α,ω*-nucleobase amide-conjugated systems and their exemplary synthetic precursors

***N*-(6-aminohexyl)-3-[5-bromo-2,4-dioxo-3,4-dihydropyrimidin-1(2*H*)-yl]propanamide (16)**

To a stirred solution of 1,6-hexylenediamine (**2c**, 3 mmol) (at room temperature) in anhydrous DMF (8 mL), a mixture containing **1c** (1 mmol), DMT-MM (1 mmol) and DMF (2 mL) was added within 1 h. Stirring was continued for 30 min. The volatiles were removed under reduced pressure using a rotary evaporator and the residue purified by gradient column chromatography (50% vol. MeOH/$CHCl_3$ to 2% $NH_{3(aq)}$/MeOH) to yield the title compound as white crystals (0.33 mmol). Mp 226–228 °C. ^1H NMR: δ = 9.20 (br s, H-3, 1 H), 8.02 (t, $^3J_1$ = 6.6 Hz, CONH, 1 H), 7.90 (s, H-6, 1 H), 5.35 (br s, NH_2, 2 H), 3.85 (t, >NCH_2, $^3J_1$ = 6.3 Hz, 2 H), 3.00 (quartet, ^3J = 6.6 Hz, CONHCH_2, 2 H), 2.65 (quintet, ^3J = 6.6 Hz, CH_2NH_2, 2 H), 2.45 (t, ^3J = 6.6 Hz, CH_2CO, 2 H), 1.58–1.10 (m, $(CH_2)_4CH_2NH_2$, 8 H). ^{13}C NMR: δ = 169.4, 162.2, 152.3, 145.2, 94.7, 53.5, 45.2, 38.4, 34.3, 30.0, 29.0, 26.1, 25.9. $C_{13}H_{21}BrN_4O_3$ (361.23): calcd. C 43.22, H 5.86, N 15.51; found C 43.05, H 6.03, N 15.51.

***N*-Boc protected amine precursor 17 and unsymmetrical conjugated α,ω-nucleosides 18–25**

General procedure:

To a stirred (room temperature) solution of the appropriate acidic component (**1b, c, e–g**) (1 mmol) in anhydrous DMF (8 mL), the amine unit (**2c, f–l, 2ba**) (1 mmol) was added. In the case of synthesis of **19**–2**3**, when amine hydrofluoroacetate was used, 1 mmol of *N*-methylmorpholine was added to liberate the free amine. When **1ea** was the acid component, an additional 1 mmol of *N*-methylmorpholine was used. After 10 minutes to the formed clear solution, DMT-MM (1 mmol) was added. The progress of the reaction was monitored by TLC (20% vol. MeOH/$CHCl_3$). After 1.5–6 h the volatiles were removed under reduced pressure

and the residue purified by gradient column chromatography (0–50% vol. MeOH/$CHCl_3$). All products were obtained as white solids.

***tert*-butyl 3-{3-[5-methyl-2,4-dioxo-3,4-dihydropyrimidin-1(2*H*)-yl]propanamido}propyl-carbamate (17)**

Mp 122–123 °C. ^{1}H NMR ($CDCl_3$): δ = 10.60 (s, H-3, 1 H), 7.34 (s, CH_2CONH, 1 H), 7.29 (s, H-6, 1 H), 5.30 (s, NHBoc, 1 H), 4.04 (t, >NCH_2, $^3J_1$ = 6.6 Hz, 2 H), 3.29 (quartet, $CONHCH_2$, $^3J_1$ = 6.9 Hz, 2 H), 3.10 (t, CH_2NHBoc, $^3J_1$ = 6.9 Hz, 2 H), 2.67 (t, CH_2CONH, $^3J_1$ = 6.6 Hz, 2 H), 1.85 (s, CH_3, 3 H), 1.62 (m, $CH_2CH_2CH_2$, 2 H), 1.42 (s, $C(CH_3)_3$, 9 H).^{13}C NMR ($CDCl_3$): δ = 170.6, 165.3, 156.4, 151.4, 142.3, 110.0, 79.2, 45.7, 37.2, 36.3, 34.9, 29.7, 28.4 (3C), 12.1. $C_{16}H_{26}N_4O_5$ (354.40): calcd. C 54.22, H 7.39, N 15.81; found C 54.40, H 7.38, N 15.76.

3-[5-methyl-2,4-dioxo-3,4-dihydropyrimidin-1(2*H*)-yl]-*N*-(9*H*-purin-6-yl)propanamide (18)

Mp 234–236 °C. ^{1}H NMR δ = 12.85 (br s, H-9, 1 H), 11.24 (br s, H-3, 1 H), 8.07 (br s, CONH, 1 H), 7.55 (s, H-2, 1 H), 7.51 (s, H-8, 1 H), 7.10 (s, H-5, 1 H), 3.81 (t, >NCH_2, $^3J_1$ = 6.6 Hz, 2 H), 2.60 (t, CH_2CO, $^3J_1$ = 6.6 Hz, 2 H), 1.74 (s, CH_3, 3 H). $C_{13}H_{13}N_7O_3$ (315.29): calcd. C 49.52, H 4.16, N 31.10; found C 49.38, H 4.03, N 30.96.

3-[5-bromo-2,4-dioxo-3,4-dihydropyrimidin-1(2*H*)-yl]-*N*-{3-[5-nitro-2,4-dioxo-3,4-dihydropyrimidin-1(2*H*)-yl]propyl}propanamide (19)

Mp 289–291 °C. ^{1}H NMR δ = 11.90–11.05 (br s, 2 H-3, 2 H), 9.33 (s, H-6, 1 H), 8.21 (t, $^3J_1$ = 6.6 Hz, CONH, 1 H), 8.10 (s, H-6, 1 H), 3.90 (t, >NCH_2, $^3J_1$ = 6.6 Hz, 2 H), 3.83 (t, >NCH_2, $^3J_1$ = 6.6 Hz, 2 H), 3.08 (quartet, CH_2NH, $^3J_1$ = 6.6 Hz, 2 H), 2.48 (t, CH_2CO, $^3J_1$ = 6.6 Hz, 2 H), 1.76 (quintet, $CH_2CH_2CH_2$, $^3J_1$ = 6.6 Hz, 2 H). ^{13}C NMR: δ = 169.7, 159.7, 155.3, 150.8, 150.2, 149.5, 145.9, 125.0, 94.1, 47.4, 45.1, 35.6, 34.2, 28.4. $C_{14}H_{15}BrN_6O_7$ (459.21): calcd. C 36.62, H 3.29, N 18.30; found C 36.77, H 3.11, N 18.21.

3-[5-bromo-2,4-dioxo-3,4-dihydropyrimidin-1(2*H*)-yl]-*N*-{6-[5-nitro-2,4-dioxo-3,4-dihydropyrimidin-1(2*H*)-yl]hexyl}propanamide (20)

Mp 133–135 °C. ^{1}H NMR δ = 12.00 (br s, H-3, 1 H), 11.77 (s, H-3, 1 H), 9.28 (s, H-6, 1 H), 8.02 (s, H-6, 1 H), 7.98 (t, $^3J_1$ = 6.6 Hz, CONH, 1 H), 3.88 (t, >NCH_2, $^3J_1$ = 6.6 Hz, 2 H), 3.82 (t, $^3J_1$ = 6.6 Hz, >NCH_2, 2 H), 3.00 (t, $^3J_1$ = 6.6 Hz, CH_2NH, 2 H), 2.44 (t, $^3J_1$ = 6.6 Hz,

CH_2CO, 2 H), 1.61 (m, >NCH_2CH_2, 2 H), 1.52–1.15 (m, $(CH_2)_3CH_2NH$, 6 H). ^{13}C NMR: δ = 169.2, 159.7, 155.0, 150.7, 150.1, 149.3, 145.9, 124.9, 94.0, 49.1, 45.2, 38.4, 34.0, 28.9, 28.3, 26.0, 25.4. $C_{17}H_{21}BrN_6O_7$ (501.29): calcd. C 40.73, H 4.22, N 16.76; found C 40.58, H 4.24, N 16.90.

3-(6-chloro-9*H*-purin-9-yl)-*N*-(3-{3-[5-methyl-2,4-dioxo-3,4-dihydropyrimidin-1(2*H*)-yl]propanamido}propyl)propanamide (21)

Mp 128–130 °C. ^1H NMR: δ = 11.24 (s, H-3, 1 H), 8.08–7.95 (m, H-2, H-8, 2 CONH, 4 H), 7.42 (d, $^4J_2$ = 0.9 Hz, H-6, 1 H), 4.34 (t, N^9(purine)-CH_2, $^3J_1$ = 6.9 Hz, 2 H), 3.81 (t, >NCH_2, $^3J_1$ = 6.6 Hz, 2 H), 3.58-3.22 (m, $CH_2CH_2CH_2$, 4 H), 2.98 (m, $NHCH_2$, 4 H), 2.66 (t, $^3J_1$ = 6.6 Hz, CH_2CONH, 2 H), 2.51 (t, $^3J_1$ = 6.9 Hz, CH_2CONH, 2 H), 1.71 (d, CH_3, $^4J_2$ = 0.9 Hz, 3 H), 1.43 (quintet, $CH_2CH_2CH_2$, $^3J_1$ = 6.6 Hz, 2 H). ^{13}C NMR: δ = 169.5, 169.1, 164.4, 156.7, 150.8, 148.3, 145.5, 141.9, 140.4, 123.9, 108.0, 56.0, 44.6, 40.0, 36.3, 35.4, 34.4, 29.1, 12.0. $C_{19}H_{23}ClN_8O_4$ (462.89): calcd. C 49.30, H 5.01, N 24.21; found C 49.25, H 4.92, N 24.06.

3-(6-chloro-9*H*-purin-9-yl)-*N*-{3-[5-methyl-2,4-dioxo-3,4-dihydropyrimidin-1(2*H*)-yl]propyl}-propanamide (22)

Mp 118–120 °C. ^1H NMR: δ = 11.24 (s, H-3, 1 H), 8.31 (t, $^3J_1$ = 6.3 Hz, CONH, 1 H), 8.05 (s, H-2, 1H), 8.02 (s, H-8, 1 H), 7.58 (d, $^4J_2$ = 0.9 Hz, H-6, 1 H), 4.36 (t, N^9(purine)-CH_2, $^3J_1$ = 6.9 Hz, 2 H), 3.56 (t, $^3J_1$ = 6.9 Hz, >NCH_2, 2 H), 3.00 (quartet, $^3J_1$ = 6.9 Hz, $CONHCH_2$, 2 H), 2.70 (t, $^3J_1$ = 6.9 Hz, CH_2CO, 2 H), 1.75 (d, $^4J_2$ = 0.9 Hz, CH_3, 3 H), 1.65 (quintet, $^3J_1$ = 6.9 Hz, $CH_2CH_2CH_2$, 2 H). ^{13}C NMR: δ = 169.3, 164.4, 156.7, 150.9, 148.3, 145.5, 141.7, 140.4, 123.9, 108.4, 54.0, 45.3, 35.7, 35.4, 28.4, 11.9. $C_{16}H_{18}ClN_7O_3$ (391.81): calcd. C 49.05, H 4.63, N 25.02; found C 49.22, H 4.55, N 24.86.

(2*S*,3*S*,5*R*)-3-hydroxy-5-[5-methyl-2,4-dioxo-3,4-dihydropyrimidin-1(2*H*)-yl]-*N*-{6-[5-nitro-2,4-dioxo-3,4-dihydropyrimidin-1(2*H*)-yl]hexyl}tetrahydrofuran-2-carboxamide (23)

Mp 106–107 °C. ^1H NMR: δ = 11.95 (br s, H-3, 1 H), 11.33 (s, H-3, 1 H), 9.28 (s, H-6, 1 H), 8.25 (t, $^3J_1$ = 6.3 Hz, CONH, 1 H), 8.12 (s, H-6, 1 H), 6.31 (dd, $^3J_1$ = 8.4 Hz, $^3J_2$ = 5.7 Hz, H-1’, 1 H), 5.60 (br s, OH, 1 H), 4.30 (br s, H-4’, 1 H), 3.83 (t, $^3J_1$ = 6.9 Hz, >NCH_2, 2 H), 3.09 (quartet, $^3J_1$ = 6.9 Hz, CH_2NH, 2 H), 2.25–2.02 (m, 2 H-2’, 2 H), 1.77 (s, CH_3, 3 H), 1.63 (m, >NCH_2CH_2, 2 H), 1.52–1.31 (m, $(CH_2)_3CH_2NH$, 6 H). ^{13}C NMR: δ = 170.1, 163.7, 158.5,

155.1, 150.7, 149.4, 137.0, 124.9, 109.4, 85.5, 73.7, 66.0, 49.2, 38.4, 28.8, 28.3, 26.0, 25.4, 12.4. $C_{20}H_{26}N_6O_9$ (494.46): calcd. C 48.58, H 5.30, N 17.00; found C 48.71, H 5.15, N 17.03.

{(2*R*,3*S*,5*R*)-3-acetoxy-5-[5-methyl-2,4-dioxo-3,4-dihydropyrimidin-1(2*H*)-yl]tetrahydrofuran-2-yl}methyl 4-[(2,4-dioxo-1,2,3,4-tetrahydropyrimidin-5-yl)amino]-4-oxobutanoate (24)

Mp 145–147 °C. ^{1}H NMR: δ = 11.39 (br s, 2 H-3, 2 H), 10.62 (s, H-1, 1 H), 9.24 (s, H-6, 1 H), 8.02 (br s, CONH, 1 H), 7.49 (s, H-6, 1 H), 6.17 (dd, $^3J_1$ = 8.4 Hz, $^3J_2$ = 5.7 Hz, H-1', 1 H), 5.19–5.17 (m, H-3', 1 H), 4.25 (d, $^3J_1$ = 4.2 Hz, H-5', 2 H), 4.15 (dd, $^3J_1$ = 6.9 Hz, $^3J_2$ = 4.2 Hz, H-4', 1 H), 2.67–2.56 (m, $(CH_2)_2$, 4 H), 2.46–2.36 (m, H-2$^{'b}$, 1 H), 2.22 (ddd, $^2J_1$ = 14.4 Hz, $^3J_1$ = 5.7 Hz, H-2$^{a'}$, $^3J_1$ = 2.1 Hz, 1 H), 2.07 (s, $OCCH_3$, 3 H), 1.79 (s, CH_3, 3 H). ^{13}C NMR: δ = 172.1, 170.2, 170.0, 163.6, 160.6, 150.4, 149.6, 135.6, 129.1, 113.3, 110.0, 83.9, 81.1, 74.0, 63.7, 35.6, 30.10, 28.7, 20.7, 12.1. $C_{20}H_{23}N_5O_{10}$ (493,42): calcd. C 48.68, H 4.70, N 14.19; found C 48.44, H 4.77, N 13.95.

3-[5-bromo-2,4-dioxo-3,4-dihydropyrimidin-1(2*H*)-yl]-*N*-({(2*R*,3*S*,5*R*)-3-hydroxy-5-[5-methyl-2,4-dioxo-3,4-dihydropyrimidin-1(2*H*)-yl]tetrahydrofuran-2-yl}methyl)-propanamide (25)

Mp 152–154 °C. ^{1}H NMR: δ = 11.71 (br s, H-3, 1 H), 11.27 (br s, H-3, 1 H), 8.17 (t, $^3J_1$ = 6.6 Hz, CONH, 1 H), 8.05 (s, H-6, 1 H), 7.48 (s, H-6, 1 H), 6.12 (t, $^3J_1$ = 8.1 Hz, H-1', 1 H), 5.28 (d, $^3J_1$ = 4.5 Hz, OH), 4.12–4.05 (m, H-3', 1 H), 3.87 (t, $^3J_1$ = 6.6 Hz, >NCH_2, 2 H), 3.73–3.67 (m, H-4', 1 H), 3.24–3.16 (m, CH_2CO, H-5', 4 H), 2.11–2.04 (m, H-2', 2 H), 1.80 (s, CH_3, 3 H). ^{13}C NMR: δ = 170.1, 169.8, 163.7, 151.2, 150.7, 147.3, 136.1, 95.4, 84.9, 83.9, 71.2, 45.1, 42.3, 41.0, 38.7, 12.0. $C_{17}H_{20}BrN_5O_7$ (486.27): calcd. C 41.99, H 4.15, N 14.40; found C 42.13, H 4.02, N 14.22.
